# Academic burnout in psychology and health-allied sciences: the BENDiT-EU program for students and staff in higher education

**DOI:** 10.3389/fpsyg.2023.1239001

**Published:** 2023-10-10

**Authors:** Lefki Kourea, Elena C. Papanastasiou, Liliana Veronica Diaconescu, Ovidiu Popa-Velea

**Affiliations:** ^1^Department of Education, University of Nicosia, Nicosia, Cyprus; ^2^Department of Medical Psychology, Faculty of Medicine, University of Medicine and Pharmacy “Carol Davila”, Bucharest, Romania

**Keywords:** stress, anxiety, mental health, intervention, prevention, university students, curriculum training

## Abstract

Studying at university involves demanding academic and clinical training requirements for students from Psychology and other health-allied fields, potentially having severe physical and mental health implications. Existing training programs for addressing burnout have focused thus far on specific areas (e.g., stress management, physical exercise, mindfulness meditation, etc.) with promising outcomes. However, no comprehensive programs have been developed to train students and staff in the early identification of burnout signs and characteristics as well as in self-assessing personal needs and habits (i.e., primary prevention), or in identifying community resources and evidence-based strategies to overcome burnout (i.e., secondary prevention). This paper describes the content development, refinement, and piloting process of the BENDiT-EU program as part of a European collaborative to address academic burnout for health-allied students. Piloting results showed that participants viewed the program positively and provided helpful suggestions for content improvement and training delivery. Future research directions should target experimental investigations of the program’s effectiveness and the longitudinal interaction of burnout with other variables (e.g., resilience).

## 1. Introduction

Studying at university entails a demanding workload (e.g., group and/or individual projects, papers, presentations, exams, attending lectures, clinical training, etc.) that may create stress and anxiety, especially if one is unable to cope with multiple academic requirements (Vizoso et al., 2019; Nyante et al., 2020). This is particularly evident for students from health-allied sciences (e.g., Medicine, Psychology, Nursing, etc.), whose clinical training involves, among others, a complex kind of patient care (e.g., Meier et al., 2001; Meim et al., 2021). Typically, the study of Medicine has been considered “difficult,” “burdensome,” or “demanding,” with many undergraduate students describing their studies as “stressful” or “very stressful” (Dahlin et al., 2005). Researchers suggest that this perception may occur early in medical students’ academic studies (Heinen et al., 2017), with up to 46.10% of undergraduate students reporting exhaustion (Almutairi et al., 2022). Many medical students describe an ever-increasing exposure to academic stress, potentially resulting in early psychiatric comorbidity (Ludwig et al., 2015).

Similar findings have been found with Psychology and Nursing students (e.g., He et al., 2018). For example, Myers et al. (2012) found that Psychology students may exhibit poor self-care habits, such as inadequate sleep, low social support, and inability to regulate emotions, while Lovell et al.’s (2015) study showed that more than 33.3% of Nursing students reported mild or severe mental illness symptoms (e.g., depression, anxiety, stress) and the most of them reported engaging in multiple unhealthy behaviors (e.g., skipping breakfast, low sleep quality, inadequate physical activity). If these early signs are not addressed adequately or diagnosed, they may eventually result in burnout syndrome (Jiménez-Mijangos et al., 2023).

## 2. Literature review – academic burnout: a three-dimensional syndrome

### 2.1. Definition and characteristics of academic burnout

As a distinct “occupational phenomenon” (World Health Organization, 2019), academic burnout consists of (1) exhaustion in an attempt to fulfill academic requirements successfully; (2) increased mental distance from one’s studies, feelings of negativism or cynicism, and (3) feelings of ineffectiveness in academic obligations (Abreu Alves et al., 2022). Its prevalence varies significantly from 8 to 71% (Ishak et al., 2013; Loayza-Castro et al., 2016), with an asymmetric self-report of sub-components (40.8–55.4% for emotional exhaustion, 31.6–35% for cynicism and 27.4–30.9% for academic accomplishment), possibly due to instrument differences, university profiles, diagnostic criteria, cultural aspects, and structure of educational systems (Frajerman et al., 2019; Rosales-Ricardo et al., 2021). Academic burnout is a cumulative phenomenon, with a higher prevalence in clinical years (Bullock et al., 2017) and tends to be under-reported. Reasons for under-reporting include insufficient peer support, stigmatization, personal stereotypes about academic endurance, or blind adherence to group norms.

A series of conceptual models were developed regarding burnout syndrome. Initially, the relationship between the three dimensions of burnout was understood as sequential, with exhaustion assumed to develop first (as a direct result of overload), followed by depersonalization (seen as a dysfunctional coping mechanism after perceiving exhaustion), and culminating with feelings of professional inadequacy and inefficacy (Maslach and Leiter, 2016). Recent models consider the perceived discrepancy between inner resources and external demands (see the Job Demands-Resources Model) (Cherniss, 1980) and the role of an ongoing, low-level loss of resources (see the Conservation of Resources model) (Buchwald and Hobfoll, 2004) as essential catalysts for burnout. Conversely, the Areas of Worklife Model offers a different perspective on burnout, as it claims that six key areas (workload, control, reward, community, fairness, and values) may contribute to burnout through the person-job imbalance (Leiter and Maslach, 2004). This model suggests that reducing work demands and increasing perceived control and adequacy of work to the employee’s values can lower the risk of burnout.

In sum, the three conceptual models guide researchers to identify ways (e.g., burnout curriculum training) to support students’ socio-emotional needs by providing self-care information and facilitating student involvement in applying work-life balance practices.

#### 2.2. Research evidence on the negative impact of academic burnout

If not acknowledged and addressed, student burnout can have significant individual consequences (Pokhrel et al., 2020; March-Amengual et al., 2022; Niedobylski et al., 2022) and high societal costs. For instance, burnout among physicians can have long-term effects on their job motivation and performance. This can directly impact the quality of life of their patients. Physicians who experience burnout may exhibit cognitive, emotional, and behavioral vulnerabilities, such as indifference, cynicism, hostility, or avoidance of targeting patients or families. If left unaddressed, these issues can lead to a significant and lasting disruption of the therapeutic relationship between physician and patient (Meier et al., 2001; Patel et al., 2018).

A series of specific factors may contribute to the emergence of burnout in health-allied academic environments. External factors typically include the significant demands of the academic program and clinical training (Hill et al., 2018), the long study duration (Radcliffe and Lester, 2003), the curriculum vastness (Satpathy et al., 2021), frequent examinations (Sreeramareddy et al., 2007), lack of perceived control (Ishak et al., 2009), massive workload (Ball and Bax, 2002), displacement from familiar support systems (Gradiski et al., 2022), and increased emotional demands of this profession (Weurlander et al., 2019). Regarding empathy, it is considered a valuable skill in all healthcare professions (Hurwitz et al., 2013). However, medical schools (especially in the clinical track) do not always prioritize empathy in their teachings (Outram and Kelly, 2014). The emotional aspect of the therapeutic relationship is often overlooked. In addition to this, personal factors like personality traits, coping ability, stress levels, and beliefs about death and suffering may influence a student’s integration into the clinical environment and their reasons for choosing this career (Popa-Velea et al., 2017).

#### 2.3. Intervention approaches to addressing academic burnout

Considering the prevalence of burnout among students and its negative consequences, prevention and intervention strategies and programs should be implemented. While many universities ensure students’ well-being and offer psychological services (e.g., counseling, career guidance, stress management, psychotherapy, etc.) (Semu, 2020), these efforts are often utilized after students have already developed burnout symptoms. This means that students may only seek support when their mental health has significantly deteriorated. Therefore, it is crucial to implement strategies and programs to prevent burnout before it becomes a problem.

According to Semu (2020), universities can help prevent burnout among Medicine and health-allied students by providing mandatory courses on the topic. These courses should be incorporated into the students’ programs of study, and should aim to raise awareness about burnout and provide intervention tools as early as possible. By doing so, universities can potentially decrease academic burnout (Reynolds, 2019). Burnout prevention courses often include cognitive behavioral training, communication skills training, relaxation exercises, counseling, and social support skills (Awa et al., 2010). Additionally, several universities have implemented optional courses on burnout prevention with encouraging results. These courses have been so successful that they have now been included in the core curriculum (Santibanez et al., 2022). However, it should be noted that offering optional courses on burnout may not be an effective universal preventative measure, as it will only be accessible to a subset of students.

Current research suggests developing and implementing comprehensive training programs that could provide holistic and targeted support for burnout (Vizoso et al., 2019). Existing training programs have focused on specific areas, such as stress management (Bragard et al., 2010), aerobic exercise (Gerber et al., 2013), and mindfulness meditation (Smith, 2014). Despite their promising outcomes, comprehensive programs have yet to be developed to address burnout (Fares et al., 2016).

## 3. Purpose

This study aimed to develop an innovative training program that provides students and staff with basic information about preventing, self-assessing, and managing burnout symptoms. The BENDiT-EU program envisions improving student well-being by (a) offering a personalized intervention plan based on student self-reflection regarding habits, needs, and responsibilities; (b) exposing participants to evidence-based intervention strategies to practice and then select suitable strategies to include in their individualized plan; (c) facilitating access to community resources, and (d) promoting inclusivity for all. Particularly, university students and staff with an international status may encounter adjustment hardships in addition to academic demands. The training content and activities have been designed to address ethnic diversity.

Furthermore, the team’s objective was to make the program sustainable with a low cost-to-benefit ratio. To achieve this, the researchers collaborated with experts and students in Medicine and health-allied fields from different European countries to ensure social and content validity in the design and evaluation of the curriculum. The training program is the result of the European-funded project “Burnout Education, Normatives and Digital Tools for European Universities” (BENDiT-EU), whose task was to construct a comprehensive set of resources for Medicine and health-allied students and professionals to address academic burnout. For more information about the project’s consortium, targets, and outcomes, readers may visit the project’s website.^1^

## 4. The BENDiT-EU training program

### 4.1. Program objectives and content

The BENDiT-EU is a comprehensive program designed to help students and staff: (a) understand the main characteristics of burnout and its progression phases; (b) self-assess any burnout signs by utilizing online psychometric measures accessed via the BENDiT-EU platform; and (c) develop an individualized intervention plan to prevent burnout signs by identifying potential risk and protective factors as well as implementing strategies from the program’s toolbox. Specifically, the BENDiT-EU program includes two training modules and a resource guide for trainers (See section 4.2) (BENDiT-EU, 2023). Table 1 describes the 30-h program’s objectives and content.

**Table 1:** The BENDiT-EU Program Objectives and Content.

Module	Training day	Participant learning outcomes	Training content	Training time
Module 1	Day 1	Define academic burnout and its main characteristics Describe the burnout progression phases Identify personal life instances of academic burnout	Exploring burnout perceptions, identifying personal activities and responsibilities that may contribute to burnout, working on case studies to identify burnout signs, consequences, and progression phases, role playing scenarios to understand the difference between stress and burnout, anxiety and burnout	6 h
	Day 2	Assess academic burnout by completing the assessment instruments via the online BENDiT-EU platform Analyze and interpret the assessment results	Connecting burnout to participant’s personal life, assessing academic burnout, navigating the online assessment platform, completing online self-assessment instruments on burnout, reflecting on the self-assessment results	5.5 h
	Day 3	Identify risk and protective factors for burnout in academic environment Describe early signs of academic burnout identification Apply early signs, protective and risk factors of academic burnout via a case study	Connecting assessment results to risk and protective factors for burnout, identifying individual, social, and organizational factors in participant’s daily life, categorizing and presenting one’s daily family, work, and personal activities in a calendar format, prioritizing daily tasks using the Eisenhower matrix, creating a personalized plan for burnout prevention	6.5 h
Module 2	Day 4	Describe available strategies for primary and secondary prevention of academic burnout Describe the strategies available for academic burnout intervention.	Practicing strategies (e.g., box breathing exercise, saying ‘no’, relaxation techniques), identifying and practicing intervention elements (health and fitness, coping skills, cognitive behavioral therapy techniques, mindfulness, self-development groups), creating a personalized toolbox based on self-guided questions	6 h
	Day 5	Map of social network based on closeness and type of support Explore campus/organizational resources for support Identify ways to address cultural diversity in academic burnout assessment and intervention materials	Defining three types of social support, creating a map of individual’s social network and listing people on the map based on certain criteria, reflecting on one’s social network map based on guided questions, defining peer mentorship and balint groups, exploring university and city resources for support	6 h

The first module is designed to provide a theoretical overview of academic burnout (Days 1–3), while the second module gives practical ideas on managing academic burnout and exploring individual and organizational resources to seek support services (Days 4–5). Specifically, Day 1 focuses on introducing the concept of burnout, exploring perceptions of burnout, and discussing its signs and symptoms. Participants engage in interactive activities such as working on case studies to identify burnout symptoms and consequences, pairing up to interview one another, discussing burnout in small groups, and completing a hands-on activity to visualize daily activities and responsibilities. Day 2 delves into academic burnout assessment. Participants learn about self-evaluation and assessment tools for academic burnout, complete assessments using the BENDiT-EU platform, and engage in reflection and discussion activities. Day 3 aims to identify ways to prevent and intervene. Participants discuss risk and protective factors for burnout, learn to identify early signs of academic burnout, and apply this knowledge through case studies. They also engage in activities designed to increase awareness of their daily routines and schedules and how these may contribute to or alleviate burnout. Day 4 centers on developing an academic burnout intervention strategy toolkit. Participants learn and practice various techniques for preventing and intervening, such as planning based on personal values, breathing exercises, relaxation strategies, cognitive behavioral therapy, and mindfulness. They also explore coping skills, social support and create an individualized “toolbox” for improving well-being and reducing academic stress. Day 5 aims to identify and discuss systems of support available to students dealing with academic burnout. Participants share their experiences with intervention strategies, learn about social support, and create a social network map. They also discuss strengthening their support networks, peer mentoring, and Balint groups.

The BENDiT-EU program is designed to be inclusive for international participants. For each training day, there are specific prompts for trainers to consider for integrating local and international students in group activities. Specifically, case study scenarios during interactive activities incorporate the experiences of international students, and participants are tasked to analyze the cases based on guided questions. Trainers ensure that international participants are fully included in groups with appropriate accommodations (e.g., language interpretation, time extension, peer pairing, assessment participation in their mother language).

### 4.2. Program supplement: the train-the-trainer resource guide

The train-the-trainer resource guide aims to equip staff from counseling or other health-related services with the necessary competencies and resources to effectively train students on burnout prevention and intervention strategies. As an additional component to the BENDiT-EU program, the guide offers essential information for preparing, delivering, and following the training sessions and practical teaching tips for adult learners.

The first section of the resource guide focuses on preparing for the training, which encompasses two key aspects: (a) an overview of adult learning principles grounded in the theory of andragogy, as proposed by Knowles (1978), and (b) preparation tactics to ensure trainers are well-versed in the content they will deliver, thereby facilitating a positive learning environment. The second section outlines guidelines for effectively conducting the training sessions, addressing elements contributing to a stimulating and conducive learning atmosphere. Topics covered include greetings, ice-breaking activities, time management, group rules, teaching strategies for active engagement, and burnout prevention resources. Lastly, the third section provides recommendations for post-training procedures and supplementary resources about burnout identification, prevention, and intervention.

## 5. The BENDiT-EU program development process

**Figure 1:**
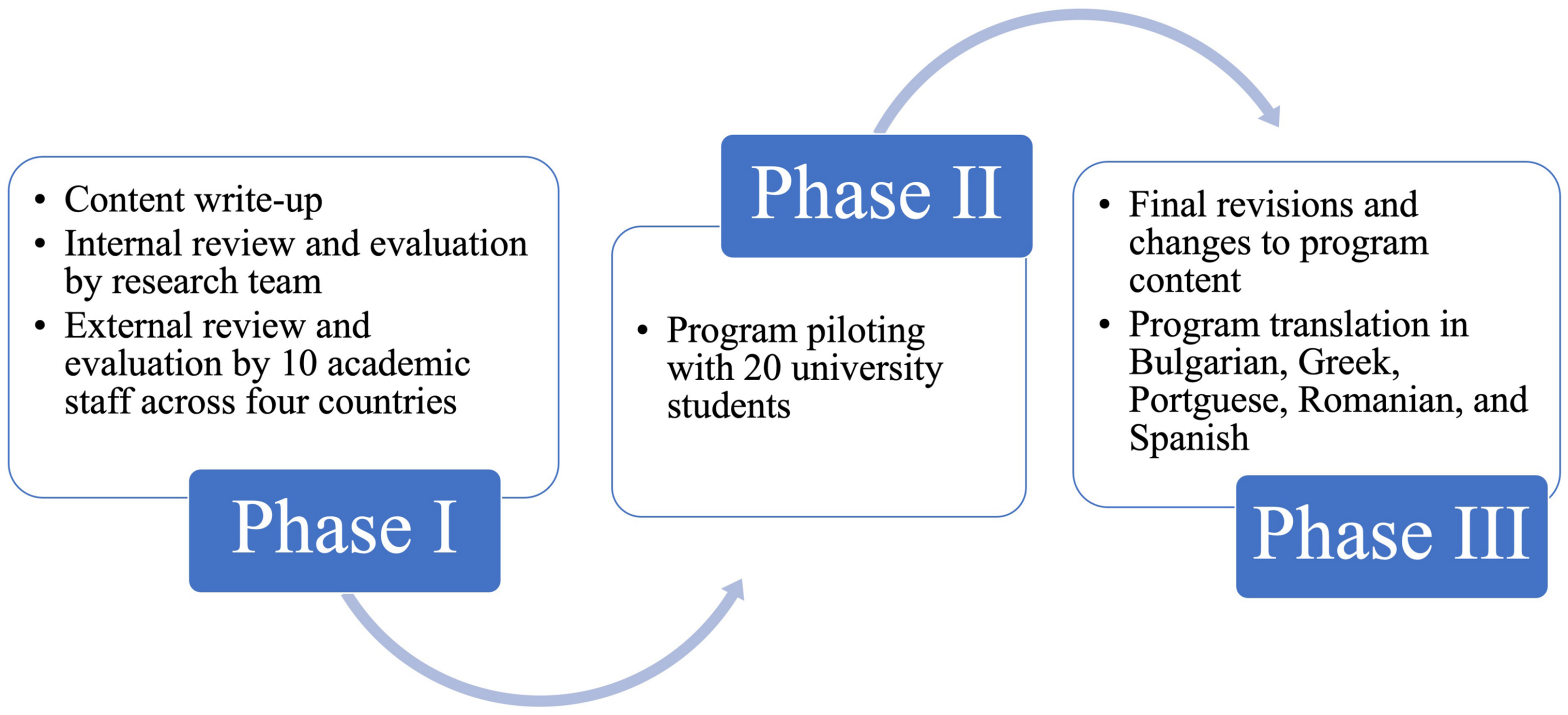
The BENDiT-EU Development Process.

As Figure 1 shows, the development and refinement of the BENDiT-EU training program followed a three-phase process to ensure the highest quality and effectiveness of the content.

### 5.1. Phase I: content development and refinement

The research team developed the program content based on an extensive literature review on academic burnout prevention and intervention (see BENDiT-EU, 2023). The content was divided into two main parts: (a) the theoretical domain with information on the characteristics, early identification, and assessment of academic burnout, and (b) the practical domain with intervention strategies and environmental supports for addressing burnout signs. Five training sessions were developed with accompanying trainer slides and student worksheets. All sessions followed a similar structure to ensure predictable and consistent implementation. Each session would start with an overview of the aim, participant learning outcomes, training materials, and allocated time. Then, an analytical description of the daily activities is presented with specific instructions for the trainee’s and trainer’s behaviors and the training materials. Every session concludes with a homework activity and a trainer prompt to ensure adaptations for culturally diverse students.

The training content was reviewed for coherence, clarity, and logical sequence by 10 research team members at an in-person meeting in Spain in July 2022. During this meeting, members were asked to present their assigned topic while soliciting colleague feedback to enhance the content and optimize the session delivery. The initial review concluded with each research member anonymously, evaluating the five training day presentations via a questionnaire and providing quantitative and qualitative feedback. Table 2 presents the quantitative feedback of the research team. As noted, more than 60% of participants evaluated the training expectations, content, and activities as very good to excellent. A third of the group rated the quality of the training content and the activities from good to very good, while half of the participants noted that the efficient use of training time was good to very good.

**Table 2:** Phase I: Internal review feedback.

	None	Fair	Good	Very good	Excellent
The training content met expectations				40%	60%
The quality of training content was satisfactory			10%	20%	70%
The quality of training activities was satisfactory			10%	20%	70%
The training time was used efficiently			20%	30%	50%

*N* = 10 members.

When analyzing the qualitative comments, there were suggestions for improving the interactiveness and engagement of training activities with the inclusion of case studies representing diverse students, incorporating prompt questions for group discussion, connecting activities with the ideas of gender and culture, including practice activity about planning and organization, and opportunities for reflection on the activities. Suggestions were provided for strengthening the training content concerning gender differences in academic burnout, factors of burnout for international students, a circle of social network support, a list of resources for international students, and summarizing the main ideas after each training day. Managing training time effectively was an additional component that was raised. Content adjustments had to be made to ensure activity transitions were kept to a minimum and time for discussion and reflection during activities was sufficient.

Following the internal review and evaluation, a revised draft was prepared and presented to 10 external academic faculty across five academic institutions in Bulgaria, Cyprus, Portugal, Romania, and Spain. The sample was purposive. The research team identified faculty who had not been involved in the project but had academic teaching experience to assess the content clarity and suitability for university students. Specifically, an assigned research member presented the modules to their colleagues at each institution, and then the latter provided qualitative feedback. All 10 participants were positioned favorably about the usefulness and necessity of the topic and its training content. They identified a clear balance between burnout theory and hands-on activities. However, four external reviewers suggested including a warm-up activity for Day 2 and making transitions more transparent at the beginning/end of each day by incorporating short revisions. The research team refined the program and then proceeded with the pilot in Phase II.

### 5.2. Phase II: content piloting

In this phase, the research team piloted the program in a five-day training with 20 undergraduate students majoring in Psychology, Medicine, Nursing, and Social Work from five countries. Students participated voluntarily and did not present any severe burnout symptoms. At the end of each day, students were asked to evaluate their satisfaction and share feedback about the program (content, activities, time allocation) and its usefulness through an anonymous survey questionnaire using paper and pencil. Project staff members blind to the study’s purpose collected the questionnaires and gave them to the research team.

Table 3 presents students’ overall experience during the pilot. As noted, more than 90% of students rated the BENDiT-EU program’s theoretical content and trainers’ delivery positively (good to very good). Based on students’ qualitative feedback, students benefitted from the introduction of self-assessment tools, time management and organization strategies, physical activity, relaxation and breathing techniques, and mapping of their social support network. Students enjoyed the interactive activities and the group discussions and acknowledged the trainers’ clear presentations. A small percentage of students (5.3%) rated the time allocation low, and 10.5% expressed low motivation and interest. In examining students’ qualitative comments, it was found that on Day 3, the assigned trainer did not allocate sufficient time to all topics and gave students more opportunities to work independently rather than in groups. As a result, students’ interest lowered for that day. Finally, students suggested some ideas for improving the training content and delivery. For instance, ideas for content improvement included providing more information about risk and protective factors and the Balint groups. Ideas for enhancing the participants’ engagement with content included role-playing the box, breathing techniques, maintaining a small team composition to allow richer discussions, and avoiding sharing responses individually in front of the whole group.

**Table 3:** Phase II: Student feedback from piloting.

	Very bad	Bad	Neither good nor bad	Good	Very good
The presentation was effective			5.3%	17.9%	76.8%
The session sparked interest and motivation		2.1%	8.4%	18.9%	70.5%
The time allocated to content was adequate		2.1%	3.2%	13.7%	81.1%
The content was well organized and systematized			1.1%	22.1%	76.8%
The knowledge provided was sufficient for daily application		1.1%	6.3%	20%	72.6%
The trainer used clear and appropriate language			5.3%	15.8%	78.9%
The trainer was knowledgeable of the content				3.2%	96.8%

*N* = 20 undergraduate health-allied students.

### 5.3. Phase III: content finalization

After receiving student feedback in Phase II, the research team revised further the program content. They then started translating the program into Bulgarian, Greek, Romanian, Portuguese, and Spanish, using the English document as their reference. As a result, the BENDiT-EU program is now accessible in six different languages.

## 6. Discussion

This paper presented the content, development, and refinement process of the BENDiT-EU program for addressing burnout in students from Psychology, Medicine, and health-allied fields. This program responds to previous research inquiries about establishing comprehensive programs (see Semu, 2020) by following a holistic approach to burnout. That is, the BENDiT-EU program (a) introduces participants to the main theoretical aspects of burnout, (b) provides practical opportunities for self-assessment of burnout, and (c) guides participants to identify concrete ways to prevent it via personalized intervention plans and toolbox strategies. Additionally, the BENDiT-EU training addresses individual differences by supporting participants from diverse health-allied fields and ethnic backgrounds, helping them to identify personal challenges, prioritize their needs, and implement effective intervention strategies.

The comprehensive focus of the BENDiT-EU program envisions training students to maintain a healthy work-life balance (Leiter and Maslach, 2004). Despite potential difficulties in the early stages of implementation, the program’s immediate and long-term benefits can outweigh them. Students, who are taught to manage burnout in their studies, can act proactively and improve their self-care and resilience skills over time. Previous studies have emphasized the need for intervention programs to support healthcare workers with low innate resilience (e.g., Nishimoto et al., 2022). By equipping new graduates with these skills, they can better handle complex and ever-changing work environments (He et al., 2018), ultimately benefiting both the healthcare industry and the public.

Another significant aspect of the BENDiT-EU program is its collaborative nature. Partner universities have pooled their knowledge, resources, and expertise to create an inclusive and theoretically-driven program. This mitigates the need for individual institutions to create distinct trainings and cultivates the ground for a future European standard in preventing academic burnout.

### 6.1. Limitations and directions for future research

As with all studies, this study presents some limitations. First, the study utilized a small sample size to establish the program’s content validity. Future research should include larger samples with diverse student populations (e.g., local vs. international students, health-allied vs. social science fields). A second limitation is the absence of experimental control to determine the program’s causality in reducing burnout. Future well-designed experimental studies could enable researchers to more accurately assess the impact of the BENDiT-EU program while controlling for potential confounding variables. Third, the program did not explore any cultural variations of burnout within the five participating countries, but it adopted the World Health Organization (2019) burnout classification. Next research directions could consider examining the cultural differences and adaptations of burnout in other European countries. Finally, this study focused solely on addressing academic burnout. Next research steps could explore variable associations, such as resilience and/or personality traits, when assessing the program’s effectiveness. For instance, screening participants on a standardized resilience measure, grouping them according to their resilience status (e.g., innate, acquired, or both), and then providing them with training opportunities would offer important conclusions about how and to what extent such training programs could strengthen participants’ resilience and reduce burnout.

### 6.2. Implications for practice

The BENDiT-EU program has significant implications for practice. First, the training content and structure are designed to cater to the needs of different student populations on campus. As stated, each participant is guided carefully to prioritize their needs and life goals and then develop a customized plan using empirically-driven intervention strategies, including some that may be applied early. Second, the program could be offered campus-wide while maintaining flexibility to adapt the program’s interactive activities and examples according to each discipline. Finally, implementing the BENDiT-EU program across campuses and countries could have notable cost-effective advantages. By adopting a universal approach through interdisciplinary collaboration, the program can potentially lessen the financial burden on campuses when dealing with students’ mental health issues (Awa et al., 2010).

## 7. Conclusion

The BENDiT-EU program provides a comprehensive approach to addressing student burnout. Unlike traditional methods focusing on isolated strategies, BENDiT-EU provides a holistic, customizable toolkit for burnout prevention and management. Its emphasis on early intervention and individualized planning could better prepare graduates for demanding professional roles while also improving the quality and cost-effectiveness of academic institutions. While more research is warranted, the program is a valuable stepping stone for future mental health interventions in academic environments.

## Data availability statement

The raw data supporting the conclusions of this article will be made available by the authors, without undue reservation.

## Ethics statement

The studies involving humans were approved by the University Research Ethics Committee/University of Nicosia. The studies were conducted in accordance with the local legislation and institutional requirements. The Ethics Committee/Institutional Review Board waived the requirement of written informed consent for participation from the participants or the participants’ legal guardians/next of kin because written informed consent had been provided as part of the ERASMUS project requirements.

## Author contributions

LK prepared the manuscript outline and performed the descriptive analysis and wrote the first draft of the manuscript. EP, LD, and OP-V contributed to the conception of the outline and wrote the sections of the manuscript. All authors contributed to the manuscript’s revision and read and approved the submitted version.

## Footnotes

^1^ https://www.bendit-eu.eu
